# Testis specific histone 2B is associated with sperm chromatin dynamics and bull fertility-a pilot study

**DOI:** 10.1186/s12958-017-0274-1

**Published:** 2017-08-01

**Authors:** Naseer A. Kutchy, Ana Velho, Erika S. B. Menezes, Marie Jacobsen, Giselle Thibaudeau, Robert W. Wills, Arlindo Moura, Abdullah Kaya, Andy Perkins, Erdogan Memili

**Affiliations:** 1 Department of Animal and Dairy Sciences, Mississippi State University, Mississippi State, MS 39762 USA; 2 Department of Animal Science, Federal University of Ceara, Fortaleza, 60040 Brazil; 3 Department of BioSciences, Rice University, Houston, TX 77005 USA; 4 Institute for Imaging Analytical Technologies, Mississippi State University, Mississippi State, MS 39762 USA; 5 Department of Pathobiology and Population Medicine, Mississippi State University, Mississippi State, MS 39762 USA; 6 Department of Reproduction and Artificial Insemination, Faculty of Veterinary Medicine, Selcuk University, 35920 Konya, Turkey; 7 Department of Computer Science and Engineering, Mississippi State University, Mississippi State, MS 39762 USA

**Keywords:** Histones, Gene ontology, Male fertility, Mammals, Sperm

## Abstract

**Background:**

Bull fertility is the degree of sperm’s ability to fertilize and activate the egg and support embryo development, and this is critical for herd reproductive performance. We used the bull as a unique model organism for the study of male fertility because cattle genetics and physiology is similar to those of other mammals including humans. Moreover, reliable fertility data along with well-established in vitro systems are available for bovine. The objective of this original study was to ascertain evolutionary diversification and expression dynamics of Testis Specific Histone 2B (TH2B) in sperm from Holstein bulls with different fertility scores.

**Methods:**

The intensity of TH2B was determined by using flow cytometry in sperm from 13 high and 13 low fertility bulls. Expression levels of TH2B were measured using immunofluorescence and Western blotting in sperm from five high and five low fertility bulls. Sequence identity, evolutionary distance and interactome of TH2B were evaluated by dotmatcher, STRING and Cytoscape. Data were analyzed using linear mixed effects model and regression plots were drawn.

**Results:**

The intensity of TH2B as measured by flow cytometry was significantly affected by an interaction between fertility group and fertility score (*P* = 0.0182). The intensity of TH2B in sperm from the high fertility group decreased (*P* = 0.0055) as fertility increased. TH2B was constantly detectable in sperm and expression levels of TH2B decreased in relation to fertility in sperm from the high fertility group (*P* = 0.018). TH2B biological functions include male gamete generation, chromosome organization, DNA packaging, DNA conformation change, chromatin organization, nucleosome organization, chromatin disassembly, spermatid nucleus elongation, spermatid nucleus differentiation, sperm motility, chromatin organization, chromatin condensation, chromatin silencing, nucleus organization, and chromatin remodeling (*P* < 0.05).

**Conclusions:**

We elucidated the cellular localization and molecular physiology of TH2B using both computational and cell biology approaches. In addition to advancing the fundamental science of mammalian male gamete, the present findings can be potentially used to evaluate semen quality and predict male fertility in the future.

**Trial registration:**

This study did not involve any live animals. We did not perform any anesthesia, euthanasia, or any kind of animal sacrifice. The cryopreserved semen samples were obtained from Alta Genetics, Inc., Watertown, WI, USA. All samples were preserved in liquid nitrogen.

## Background

Spermatogenesis is the development of mature male germ cells in the testes capable of forming highly differentiated sex cells called spermatozoa. During mammalian spermatogenesis, complex biochemical and morphological transformations occur in the sperm head. The nucleus of a sperm progressively differentiates, undergoing chromosomal condensation, synapsis, genetic recombination, and extensive chromatin reorganization [1, 2]. During this chromatin reorganization, occurring in the testis, basic histones are replaced by transition nuclear proteins, (TNPs) and then by protamines [3]. Such displacement of nuclear proteins causes transformation in the shape of sperm head [4], hydrodynamicity and chromatin compaction, both of which are important for sperm motility and fertilization [5]. Replacement of canonical histones by testis specific histones, for example histone 2B (H2B), to testis specific histone 2B (TH2B) causes relaxation of the histone-bound chromatin by increased eviction of canonical histones from sperm [6]. Homology of somatic histone (H2B) to testis specific (TH2B) in human sperm is 85% [7].

Transition of H2B to TH2B sets nucleosome stability, ensuring a genome-wide change of nucleohistone to intermediate structural entities, which in turn are required for the assembly of TNPs (TNP1 & TNP2) and protamines (PRM1) [6]. TH2B induces open chromatin structure and plays a role in inter-nuclear protein replacement in sperm chromatin [8]. Chromatin remodelers, chromodomain helicase DNA binding protein 5 (CHD5), and bromodomain-containing protein 4 (BRD4) aid TH2B during nuclear protein replacement in the sperm chromatin. While CHD5 is a member of the chromodomain helicase DNA binding (CHD) family and regulates sperm chromatin structure [9], BRD4 belongs to the bromodomain-containing protein family that regulates transcription of genes in sperm by binding to hyperacetylated genomic regions [10]**.**

The octamer-shaped nucleohistone complex consists of histones H2A, H2B, histone 3 (H3) and histone (H4), all involved in sperm DNA packaging. Along with protamines, they form a tightly coiled and compacted structure known as the toroidal model [11]. Retained sperm histones are vital for the histone-bound genes activated before and**/**or after fertilization. Defects in sperm chromatin are linked to spontaneous abortion and failures of assisted reproductive techniques in human [12, 13]. These defects include disrupted DNA integrity due to mutations, and apoptotic DNA fragmentation as a result of exposure to environmental agents and free radicals [14, 15]. Such disruptions of chromatin proteins, transcriptional factors, and aberrant histone methylation have been speculated to contribute to the decreased fertility in human [16, 17]. Abnormal protamine to histone ratios cause infertility [18]. Therefore, proper TH2B to protamine ratios are important for efficient packaging of sperm chromatin [6]. Functional significance of TH2B in the oocyte remains unclear, and the maternal TH2B might be involved in the release of protamines from sperm following fertilization of the egg, thereby facilitating incorporation of maternal histones into the chromatin of developing embryo [8, 19].

Abnormal retention of histones in the sperm is an indication of immature sperm, and abnormal ratios of protamine to histone cause infertility [18]. Therefore, TH2B is a potential biomolecular marker useful for analysis of semen quality, potentially predicting bull fertility and sire’s suitability for artificial insemination (AI). Better elucidation of the dynamic role of TH2B advances the understanding of its chromatin remodeling role in bull sperm. The present study was conducted to test the central hypothesis that different cellular intensities, expression levels, and localization of TH2B are associated with sperm chromatin dynamics and bull fertility. Bovine TH2B was localized in sperm head and its expression levels (*p* = 0.0055) and intensity (*p* = 0.018) were lower in sperm from the high fertility bulls as compared to low fertility ones. Protein sequence of TH2B is conserved among human, rat, mouse and bovine. Further, TH2B has significant gene ontology terms for molecular functions in chromatin and DNA binding and biological functions in sperm development differentiation and motility, nucleosome assembly, chromatin remodeling and spermatid nucleus differentiation (*P* < 0.05). Because of the genetic and physiological similarities among mammals [20–23], the findings of the present study advance fundamental science of mammalian development and reveal potential biomolecular markers for semen evaluation and fertility prediction.

## Methods

### Experimental design

Cryopreserved semen straws from Holstein bulls (Alta Genetics, Inc., Watertown, WI, USA) were used in the present study. Straws from 26 bulls were used for the flow cytometry experiment (Table. 1) and 10 bulls were used for immunofluorescence and Western blotting assays (Table. 2). Bulls are considered fertile if they repeatedly mount to serve females and bring about 90% pregnancies in 50 females in a 9-weeks time [24]. Therefore, high fertility (HF) bulls have 80–90% pregnancies in 50 females in a 9-weeks time, whereas low fertility (LF) bulls have 70–80% pregnancies in 50 females in a 9-weeks time and less than 40% is sub fertile. In our study, the bulls were divided as HF and LF as reported in detail in our previous publications [25, 26]. Non-return rate (NRR) on day 40 after insemination were calculated for all bulls and the fertility score was ranked as the deviation of each NRR from the average. In the present study, we grouped the bulls with positive deviation from average NRR as HF and those animals with negative deviation from average as LF. Fertility data of the selected bulls are periodically updated with information from partnering herds [25]. Then, we tested the hypothesis that different cellular intensities, expression levels, and localization of TH2B are associated with sperm chromatin dynamic and bull fertility using methods in immunofluorescence, Western blotting, flow cytometry and bioinformatics.

**Table 1:** Fertility phenotypes of the Holstein bulls used for flow cytometry analysis

High fertility bulls			Low fertility bulls		
Bull number	No of breeding’s	In vivo fertility score	Bull number	No of breeding’s	In vivo fertility score
1	705	3.9	14	974	−4.7
2	965	3.7	15	555	−4.2
3	2924	3.6	16	1083	−4.1
4	737	3.5	17	793	−4.1
5	3137	3.4	18	758	−4.1
6	859	3.4	19	862	−4.1
7	745	3.1	20	469	−4.1
8	2195	3	21	892	−3.8
9	1134	3	22	987	−3.7
10	965	3	23	1105	−3.7
11	682	2.9	24	901	−3.6
12	3647	2.8	25	927	−3.6
13	6051	2.6	26	1020	−3.6

Bulls 1–13 are designated as the HF group and bulls 14–26 are designated as the LF group. All bulls were individually represented with their in vivo fertility scores and the number of breeding’s as well as no significant differences in their sperm parameters. Fertility scores are expressed as the percent deviation of each conception rate from the average conception rate of all the bulls as previously described [26]

**Table 2:** Fertility phenotypes of the Holstein bulls whose sperm were used for immunofluorescence and Western blotting

High fertility bulls			Low fertility bulls		
Bull no	No of breeding’s	In vivo fertility score	Bull no	No of breeding’s	In vivo fertility score
1	965	3.7	6	867	−4.9
2	1026	3.7	7	974	−4.7
3	2924	3.6	8	555	−4.2
4	3137	3.4	9	793	−4.1
5	859	3.4	10	1083	−4.1

Bulls 1–5 are designated as the high fertility (HF) group and bulls 6–10 are designated as the low fertility (LF) group. All bulls were individually represented with their in vivo fertility scores and the number of breeding’s. Fertility scores are expressed as the percent deviation of each conception rate from the average conception rate of all the bulls as previously described [26]

### Evaluation of TH2B expression in bull sperm by flow cytometry

Flow cytometry experiments were performed according to methods described by Dogan et al. (2015) [27] and Odhiambo et al. (2011) [28], with modifications. Flow cytometry was used to quantify the TH2B cell population by passing individual sperm through a laser beam of the appropriate wavelength. Following settings for BD-FACSCalibur flow cytometer (BD Bioscience San Jose, CA 95131–1807, USA), were used; laser line 488 nm, emission filters 530/30 nm and fluorochrome 542 nm. TH2B molecules were conjugated to fluorescent antibodies to detect and quantify the presence of this protein. Semen straws from 26 Holstein bulls, 13 high fertility (HF) and 13 low fertility (LF), were removed from liquid nitrogen and thawed at 37 °C for 30 s. The extenders were then separated from the cells by centrifugation at 2000 x *g* at 4 °C for 5 min. And pellets were washed twice in washing buffer (WB: PBS with 0.1% Bovine Serum Albumin BSA) and again centrifuged at 2000 x *g* at 4 °C for 5 min. The pellets were then fixed in 1 ml of 4% formaldehyde at RT for 1 h in separate centrifuge tubes. The samples were then centrifuged at 3000 x *g* at 4 °C for 5 min and pellets were resuspended in 250 μl of PBS and immediately permeabilized in 250 μl of 0.1% Triton X-100 in 0.1% sodium citrate in PBS on ice for 2 min. The pellets were resuspended in 500 μl of PBS, filtered through a flow cytometric tube using a cell strainer cap (Becton Dickinson Labware; catalogue no. 352235), and then incubated with the primary antibody at 4 °C overnight. Primary antibody was TH2B (Rabbit polyclonal to Testes Specific Histone H2B; Abcam, Cambridge, MA, USA; catalog # 23913; 1/250 dilution). Next day, samples were centrifuged at 3000 x *g* at 4 °C for 5 min, washed once in 500 μl of washing buffer, centrifuged at 3000 x *g* at 4 °C for 5 min and incubated with secondary antibodies for 2 h at RT. The secondary antibody was donkey anti-rabbit IgG-FITC (Santa Cruz, Dallas, Texas, USA; catalog # 2090; 1/250 dilution). Following the incubation, the samples were washed twice in WB (3000 *g* at 4 °C for 5 min). Sperm samples were then analyzed using the BD-FACSCalibur flow cytometer (BD Bioscience San Jose, CA 95131–1807 USA).

### Visualization of sperm TH2B using Immunofluorescence

Immunofluorescence was performed according to the methods described by Li et al. (2008) [29] and de Oliveira et al. (2013) [26], with modifications. Briefly, cryopreserved semen straws from five high fertility and five low fertility bulls were thawed in a water bath at 37 °C for 30 s (sec). Sperm samples were washed with PBS containing protease inhibitors (cOmplete; Roche, Indianapolis, IN, USA; catalog # 04693116001), and 10 mM ethylenediaminetetraacetic acid (EDTA). Then, the solution was centrifuged at 2000×*g* at room temperature (RT) for 5 min (min). In addition, the sperm pellets were incubated with 20 mM CHAPS at RT for 20 min. Sperm chromatin was then decondensed in 10 mM DTT and 1 mg/ml of heparin at RT for 30 min [30]. Moreover, sperm were fixed in 4% paraformaldehyde at 4 °C for 10 min. Following fixation, cells were permeabilized with 0.2% Triton X-100 and 0.1% bovine serum albumin (BSA) in PBS at RT for 15 min. Sperm were then washed in 50, 70, 95 and 100% ethanol at RT for 1 min each. The excess ethanol was removed by quick decanting followed by an additional step of fixation using 100% methanol at −20 °C for 20 min. Excess methanol was removed using washing buffer (WB: PBS containing 0.1% Triton X-100) and the sample was blocked with 1% BSA in the WB at RT for 1 h (h). Sperm were probed with primary antibodies against TH2B (Rabbit polyclonal to Testes Specific Histone H2B; Abcam, Cambridge, MA, USA; catalog # 23913; 1/200 dilution) at 4 °C overnight followed by a washing step and probing with secondary antibody of donkey anti-rabbit IgG-FITC against TH2B (Santa Cruz, Dallas, Texas, USA; catalog # 2090; 1/5000 dilution) at RT for 1 h, and then with 2.5 mg/ml of DAPI at RT for 10 min. Coverslips were placed onto the slides using a drop of an antifade mounting medium (VECTAshield, H-1000) and sealed using a nail polish border. The samples were examined under a confocal fluorescence microscope (Zeiss LSM 510) under 40X and 63X magnifications using immersion oil. The experiments were repeated three times and the data were statistically analyzed.

### Extraction of nuclear sperm protein for western blotting

Sperm TH2B was extracted according to the methods of Aoki et al. (2005) [31] and de Oliveria et al. (2013) [26], with some modifications. Briefly, cryopreserved semen straws from five high fertility and five low fertility bulls were thawed at 37 °C for 30 s and washed twice in PBS with protease inhibitor, centrifuging each time at 700×*g* at 4 °C for 5 min. An aliquot containing 25–40 × 10^6^ sperm was used to extract the nuclear proteins. In order to lyse the cells, sperm samples were washed twice with 400 μL of 1 mM Phenyl methylsulfonyl fluoride (PMSF) in ddH_2_O, centrifuged each time at 700×*g* at 4 °C for 5 min. Then, 100 μL of 20 mM EDTA, 1 mM phenylmethylsulfonyl fluoride (PMSF), and 100 mM Tris (pH 8.0) were added to the pellets followed by the addition of 100 μL of 6 M guanidine hydrochloride, 575 mM dithiothreitol (DTT), and 200 μL of 552 mM iodoacetamide. The samples were protected from light and incubated at 20 °C for 30 min. In addition, the samples were supplemented with 1 mL of cold ethanol (−20 °C), and each sample was then incubated at −20 °C for 1 min and centrifuged at 12000×*g* at 4 °C for 10 min. The ethanol wash was repeated once more and the pellet was resuspended in 1 mL of 0.5 M HCl and incubated at 37 °C for 15 min and centrifuged at 10,000×*g* at 25 °C for 10 min. The supernatant was kept and the nuclear proteins were precipitated by the addition of 300 μL of 100% trichloroacetic acid (TCA) to a final concentration of 20% TCA. The solution was incubated at 4 °C for 5 min and centrifuged at 12000×*g* for 10 min. The pellet was washed twice in 500 μL of 1% 2-mercaptoethanol in acetone. The final pellet was dried out and stored at −30 °C.

### Western blotting analyses of sperm nuclear proteins

Protein concentration in samples containing sperm protein extracts was determined in triplicates using Quick Start™ Bradford Protein Assay Kit 2 (Bio Rad®, Hercules, CA, USA; catalogue # 5000202). In addition, sperm nuclear proteins were precipitated with cold acetone at −20 °C for 3 h, followed by centrifugation at 10,000×*g* at 4 °C for 10 min. The supernatants were discarded and the pellets were resuspended in 50 μL of 1× Laemmli sample buffer (Bio Rad®, Hercules, CA, USA) with 5% 2-mercaptoethanol, vortexed (10 s), boiled for 10 min, and stored −30 °C. An aliquot of sperm nuclear proteins (10 μg) was reduced, denatured and separated with a vertical polyacrylamide gel electrophoresis (4–20% SDS-PAGE; Mini-Protean TGX™ gel). Protein bands were transferred from the gels to an Immobilon®-P polyvinylidene difluoride (PVDF) membrane using HEP-1 semi-dry electro blotting (Thermo-Scientific Inc.®; Waltham, MA USA) set at 46 mA for 2.5 h. Binding sites were blocked with 5% BSA in PBS-0.1% Tween 20 (PBS-T) at RT under mild agitation for 1 h, followed by an incubation with primary antibodies against bovine TH2B (Abcam®, Cambridge, MA, USA; 1/1000 dilution; rabbit polyclonal IgG; catalog #23913). Lamin B1 was used as internal loading control (Abcam®, Cambridge, MA, USA 1: 2000, rabbit polyclonal IgG; catalog #16048; Abcam) in PBS-T with 1% of BSA at RT for 1 h. Because, the expression levels of Lamin B1 were not stable, therefore another protein band (~25 KDa) served as second internal control. Membranes were then washed three times for 10 min in PBS-T and incubated with secondary antibodies (1:10,000; donkey ant-rabbit IgG-HRP; sc-2313; Santa Cruz Biotechnology®) and Precision Protein™ StrepTactin-HRP Conjugate (Bio Rad®, Hercules, CA, USA; 1/10,000; catalog # #1610380) in PBS-T with 1% of BSA at RT for 1 h. Membranes were washed with PBS-T three times for 5 min each. The bands were revealed using a chemoluminescence reagent (Clarity™ Western ECL Substrate, Bio-rad®, Hercules, CA, USA) and Image Laboratory software (Bio-Rad®) for 30 s. The band intensity of TH2B histone protein was analyzed using ImageJ 1.x [32].

### Analyses of sequences and interactions of TH2B using bioinformatics

Emboss dotmatcher was used to compare protein sequences of predicted bovine TH2B with human TH2B, bovine H2B with human H2B, and bovine TH2B with bovine H2B (http://www.bioinformatics.nl/cgi-bin/emboss) to ensure the sequence similarity of bovine TH2B for downstream computational biology analysis. Protein sequences of predicted TH2B and H2B of bovine were compared using computational tools. Protein sequences of TH2B and H2B were first extensively searched and aligned using UniProt for the bovine (www.uniprot.org, UniProt, 2012). The sequence alignment was performed using Clustal Omega method and the percent identity matrixes between the sequences were obtained (http://www.ebi.ac.uk/Tools/msa/clustalo/).

Predicted protein-protein interactions among TH2B, H2B, H2A, H3, H4 TNP1, TNP2, PRM1, BRD4 and CHD5 of bovine, human and mouse were obtained using STRING database (Search Tool for the Retrieval of Inter-Acting Proteins; http://string-db.org/) [33]. The testis specific histone 2B (TH2B) in mature sperm interacts with chromatin proteins H2A, H3, H4, H1, TNP1, TNP2, PRM1, BRD4 and CHD5. Thus, the interactome of *TH2B* using the biological networks gene ontology tool (BiNGO) within Cytoscape 3.3 (http://www.cytoscape.org) was elucidated as well [34]. To analyze the interactome of *HIST1H2B (TH2B)*, a merged-network was generated in Cytoscape by entering the query keywords “*HIST1H2B* for *TH2B*”, “*H2A*”, “*H3*”, “*H4*”, “*TNP1*”, “*TNP2*”, “*PRM1*”, “*PRM2*”, “*H2B*”, “*BRD4*” and “*CHD5*” into the search bar and results were retrieved from the BiNGO plugin to assess overrepresentation of GO categories.

### Statistical analysis for flow cytometry, western blotting and immunofluorescence

The associations between in vivo fertility scores of bulls with sperm TH2B intensity (measured by flow cytometry) and expression levels of TH2B (estimated by Western blotting) were assessed using mixed model analysis with PROC MIXED from SAS for Windows 9.4. For this mixed model, in vivo fertility group was the fixed effect. The random statement included replicate within repeat, repeat within bull, and bull as random effects. The relationship between fertility score and TH2B intensity was assessed through mixed model linear regression using PROC MIXED with SAS for Windows 9.4. In an initial model, fertility score, fertility group, and their interaction term were included as fixed effects. The random statement included replicate within repeat, repeat within bull, and bull as random effects. Fitting this model indicated no significant interactions between fertility score and fertility group, but the fertility scores were widely separated between the two groups. Accordingly, separate models with fertility score as the fixed effect were fit for both the high and low fertility groups to better understand the relationships between fertility and TH2B intensity. Regression line plots were developed using SGPLOT with SAS for Windows 9.4. The distribution of the conditional residuals was assessed to ensure that assumptions for the statistical models have been met. An alpha level of 0.05 was used to determine statistical significance.

## Results

### Expression dynamics of TH2B in bull sperm using flow cytometry

**Fig. 1:**
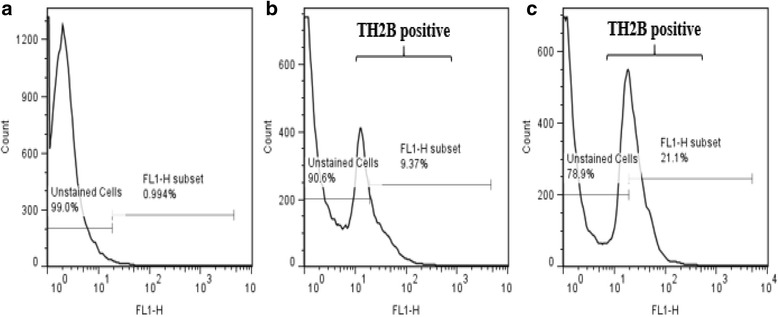
Differential expression of TH2B in sperm from bulls with different fertility scores. The negative control showing the absence of TH2B fluorescence in the unstained sperm (**a**). Percentage of sperm expressing TH2B and unstained in high fertility bulls (**b**). The percentage of sperm in low fertility bulls expressing TH2B positive and unstained cells (**c**). The percentage of sperm in FL1-H subset indicates expression of TH2B

**Fig. 2:**
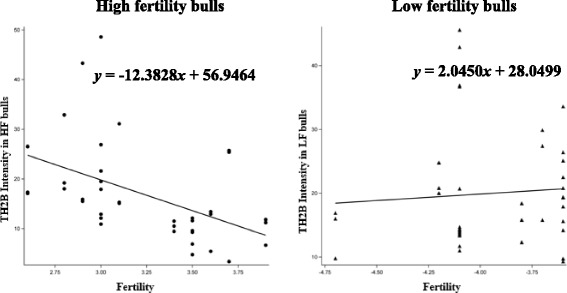
Regression models showing in the intensity of TH2B in sperm as related to fertility scores in bulls. The regression line was determined using the model predicted intensity values for each value of fertility score using the mixed effects model. A scatter plot of unadjusted data points was superimposed on the regression line plot. Regression equations are shown for high fertility (*y* = −12.3828*×* + 56.9464) and low fertility (*y* = 2.0450*×* + 28.0499) bulls

Total of 150,000 sperm per each bull were analyzed to investigate the expression of testis specific histone 2B (TH2B) in sperm from 13 high fertility and 13 low fertility bulls (Table. 1). Flow cytometric measurements of sperm TH2B showed different histogram profiles between HF and LF bulls (Fig. 1). As evaluated by regression analysis, the interactions between TH2B intensity and fertility scores of bulls were significant (*P* = 0.0182). The expression levels of TH2B was highly significant compared to fertility score in the high fertility group (*P* = 0.0055; *y* = −12.3828*×* + 56.9464) but no significance was found in the low fertility group (Fig. 2; *y* = 2.0450*×* + 28.0499).

### Cellular localization of TH2B in bull sperm using immunofluorescence

**Fig. 3:**
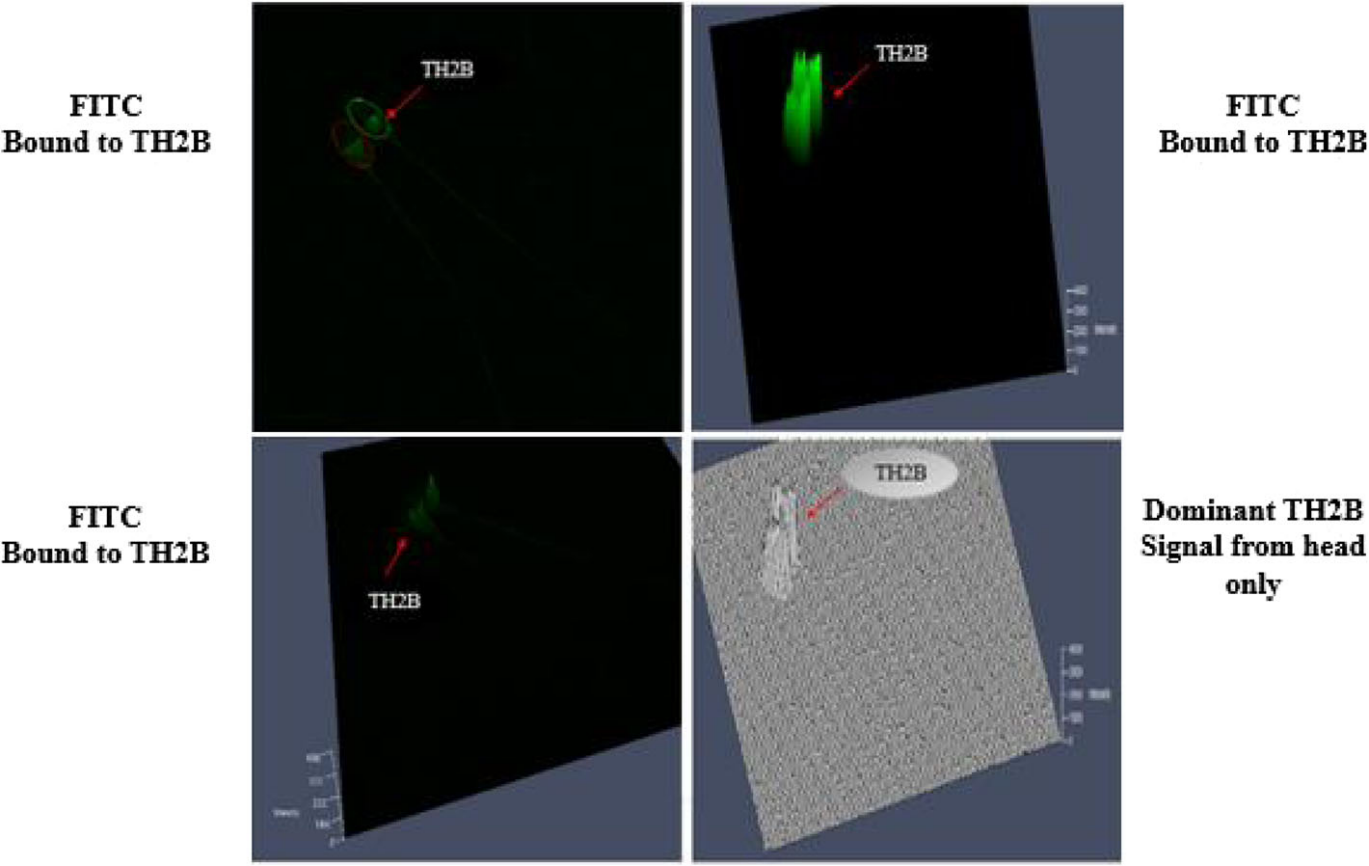
Cellular localization of TH2B in bull spermatozoa. The intensity of FITC bound to TH2B protein (**a**, **b** and **c**). Dominant signal of TH2B coming from sperm head, depict merged images of two sperm using confocal microscope, showing expression dynamics and localization of TH2B in head of bovine spermatozoa (**d**)

**Fig. 4:**
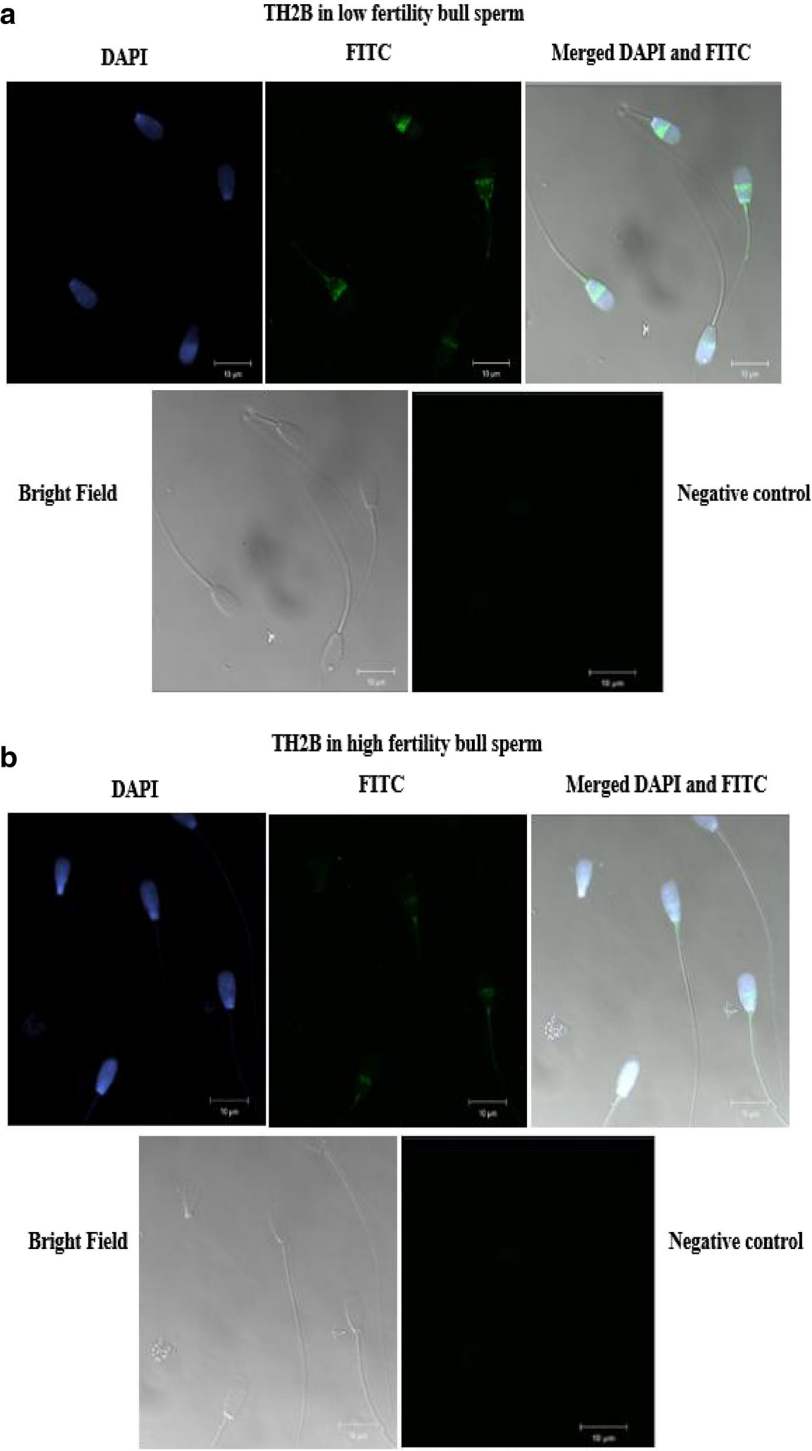
Immunostaining of TH2B in low and high fertility bull spermatozoa. Intensity of TH2B in sperm from low (**a**) and high fertile bulls (**b**) using confocal microscopy. DNA stained with DAPI (*blue*) in a bull sperm; TH2B histone linked to FITC-conjugated secondary antibody (*green*) in bull sperm; merged images of DAPI and FITC; bright field images; and negative controls

We determined the cellular localization of TH2B in sperm of low vs. high fertile bulls using immunofluorescence and confocal microscopy.TH2B signal was detectable in sperm head, concentrated over the equatorial and subacrosomal area of sperm. The signal was visualized as a band just beneath the acrosome, with a clear space between the base of the band and the proximal end of tail of sperm (Fig. 3). The immunofluorescence signal for TH2B was brighter in sperm of the low fertile than that of the high fertile bulls (Fig. 4; Table 2). As evaluated by regression analysis, association between TH2B and bull fertility was not different. The fact that the signals measured by immunofluorescence were obtained from 45 spermatozoa per bull could be the reason for the non-significance.

### Expression of TH2B in bull sperm using western blotting

**Fig. 5:**
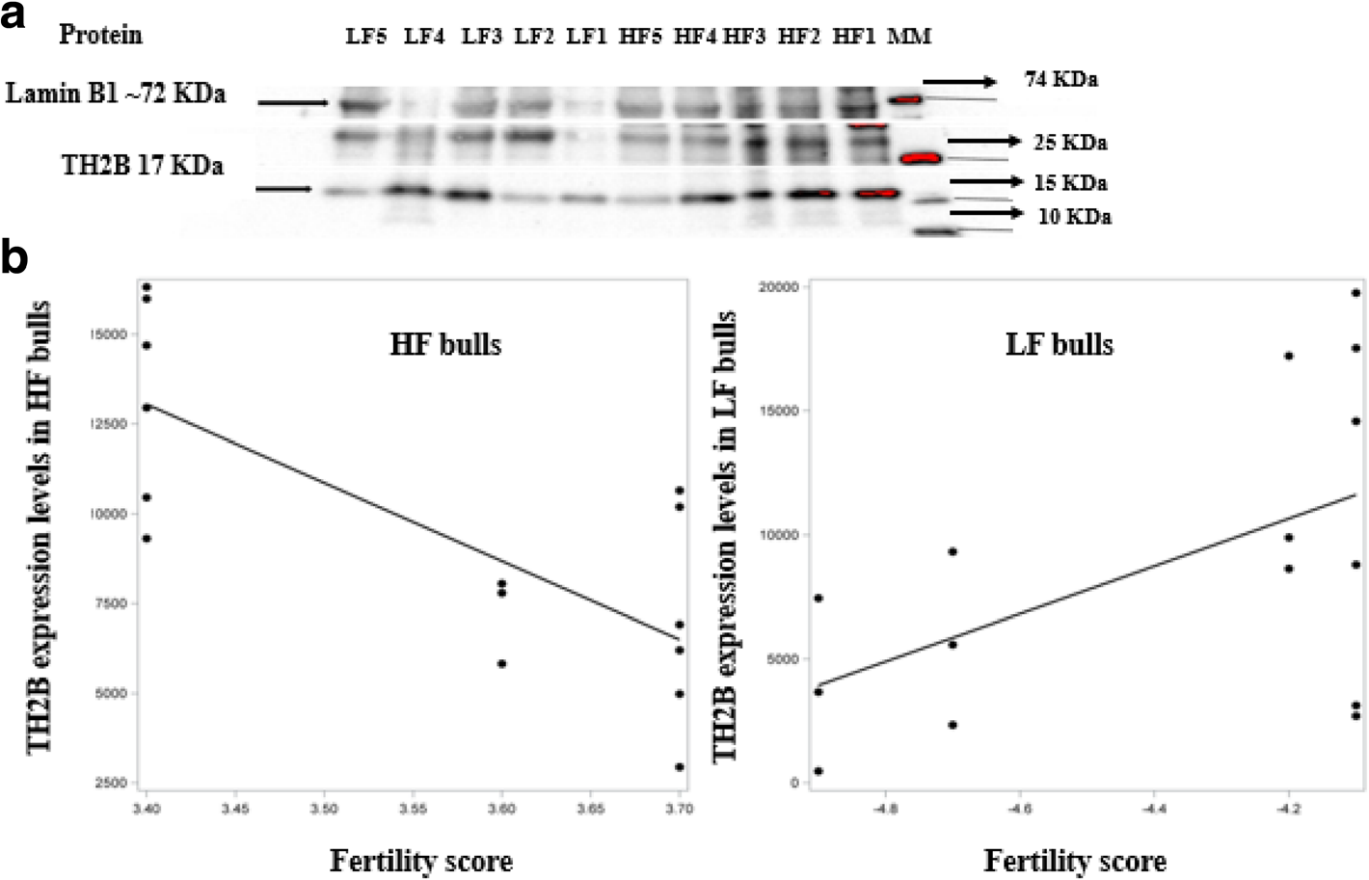
Detection of TH2B protein using Western blotting and regression models showing variation in the intensity of TH2B in sperm as related to fertility scores in Holstein bulls. **a** MM stands for molecular markers; LF1–5 and HF1–5 refer to samples from low vs. high fertility bulls, respectively. The same amounts of nuclear proteins were loaded into each lane. As TH2B is a nuclear protein and the expression levels of our internal control (Lamin B1) were not stable, another protein band (~25 KDa) served as the internal control. **b** The regression line was determined using the model predicted intensity values for each fertility score using the mixed effects model. A scatter plot of unadjusted data points was superimposed on the regression line plot. Regression equations are shown for high fertility (*y* = −22,315*×* + 89,049) and low fertility (*y* = 9603*×* + 50,993) bulls

An immunoblotting approach was used to detect the expression of TH2B in the sperm from low vs. high fertility bulls. TH2B protein band intensity was analyzed using ImageJ [32]*.* Our results showed that the TH2B was consistently detectable in sperm from all of bulls (Fig. 5). The expression levels of TH2B were significantly affected by an interaction between fertility score and fertility group (*P* = 0.0058). In high fertility bulls, as fertility decreased, TH2B expression increased (*P* = 0.0182; *y* = −22,315*×* + 89,049) signifying that the bulls with the highest fertility had the least TH2B retention in their spermatozoa. In low fertility bulls, as fertility decreased, TH2B expression decreased (*P* = 0.0737; *y* = 9603*×* + 50,993; Fig. 5) indicating that the bulls with the lowest fertility had the highest of retained TH2B in their spermatozoa.

### Bioinformatic analyses of TH2B and H2B

**Fig. 6:**
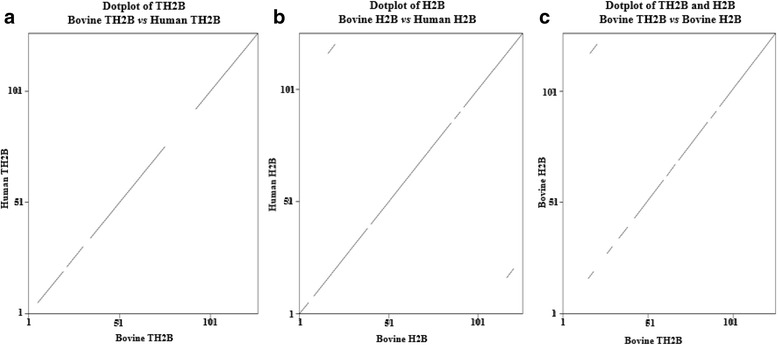
Threshold dotplot of TH2B and H2B between bovine and human. The emboss dotmatcher of predicted TH2B of bull vs. TH2B of human is a perfect diagonal line with two frame shift mutation sites (**a**). The emboss dotmatcher of H2B of bull vs. H2B of human revealed a low complexity region between bovine H2B and human H2B (**b**). Dotmatcher of TH2B of bull vs. H2B of bull showed two areas of different amino acids (**c**)

Histone variant of TH2B was identified as the testis specific H2B [35, 36]. TH2B is encoded by the gene *HIST1H2B* [37]. Mouse *TH2B* gene as well as TH2B protein have been sequenced [19], and a predicted sequence of bovine *TH2B* gene *HIST1H2BA* (http://www.ncbi.nlm.nih.gov/nuccore/XM_010825421.2) has been used in our study, because bovine *TH2B* gene as well as TH2B protein have not been sequenced yet. Therefore, the predicted gene *HIST1H2BA (TH2B)* had 85.7% similarity with known bovine *H2B,* using multiple sequence alignment method Clustal Omega (http://www.ebi.ac.uk/Tools/msa/clustalo/) [38]. Comparisons among predicted TH2B protein sequences in bovine and human revealed higher similarity patterns of diagonal lines (Fig. 6). The comparison of predicted bovine TH2B sequence with human TH2B showed that the match was a perfect diagonal line with three frame shift mutation sites, which might have resulted in change in sequence of amino acids (Fig. 6). Similarly, comparison between bovine H2B and human H2B showed the match was a perfect diagonal line having four frame shift mutation sites, resulted change in sequence of amino acids (Fig. 6). The sequence comparison of bovine TH2B with bovine H2B showed two areas of different amino acids, due to frame shift mutations resulting in different amino acid sequences (Fig. 6) [39].

### Interactions among sperm nuclear proteins from bovine, human and mouse

**Fig. 7:**
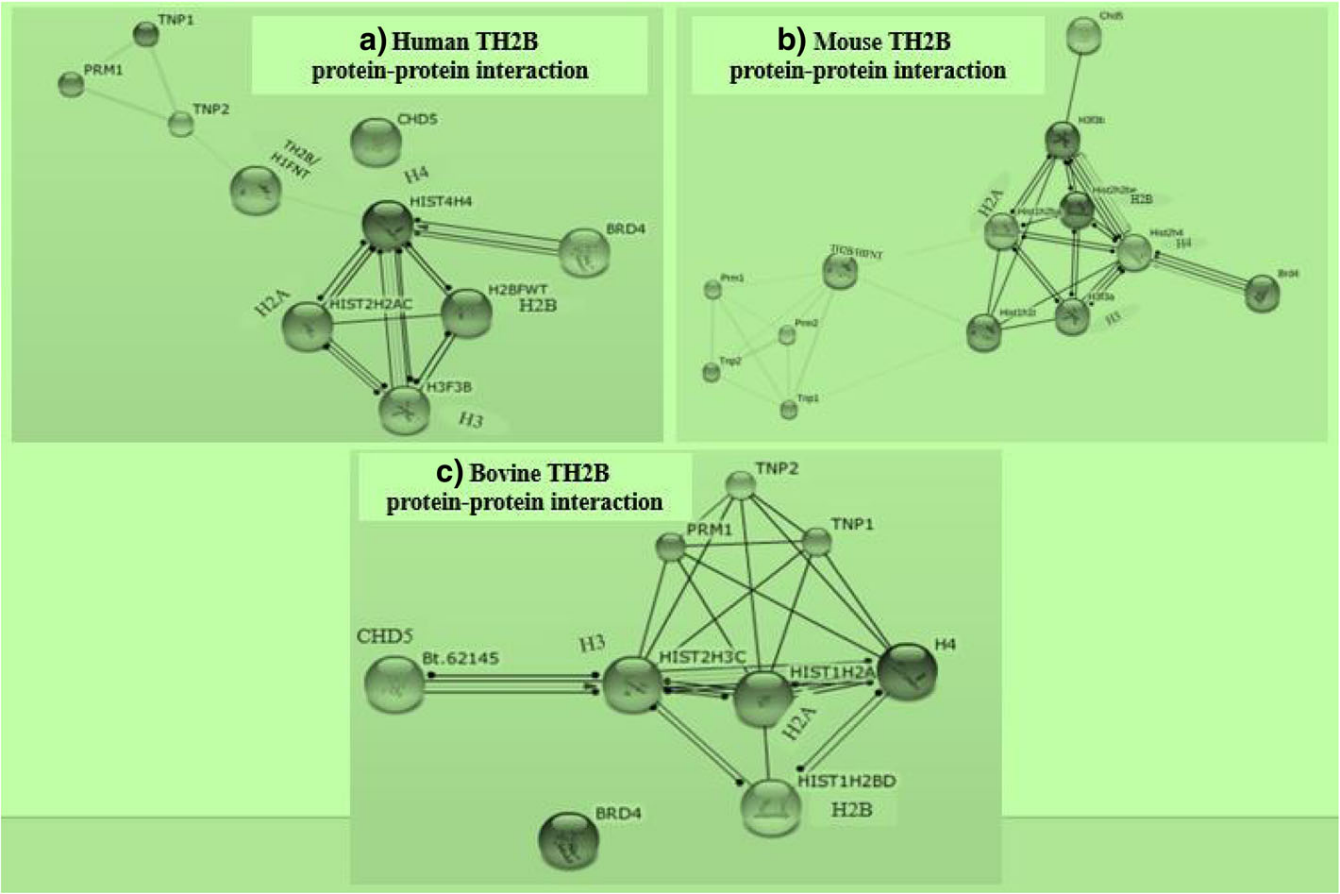
Protein-protein interactions involving TH2B. TH2B/H1FNT involved in replacement of TNPs by protamines in human and mouse are depicted in (**a**; **b**) and the missing link with possibly same role in bovines (**c**)

We used the STRING database to depict the interactions of sperm proteins in human, mice, and bovines respectively. Analyses of the interactions of nuclear proteins of developed using STRING for human, mouse, and bovine (Fig. 7) showed the missing link as TH2B; which functions in mediating the transition of nuclear proteins from histone to protamine in bovine spermatozoa. In humans and mice, TH2B is known to interact with chromatin remodelers (BRD4 and CHD5) and is involved in replacement of histones with protamines but function(s) of bovine TH2B has yet to be discovered. However, we found the high prediction of likeliness that bovine TH2B is also involved in eviction of retained histones and replacement of those with highly basic protamines, while assisted by two chromatin remodelers BRD4 and CHD5 (Fig. 7).

### Interactome of testis specific histone 2B (TH2B)

**Fig. 8:**
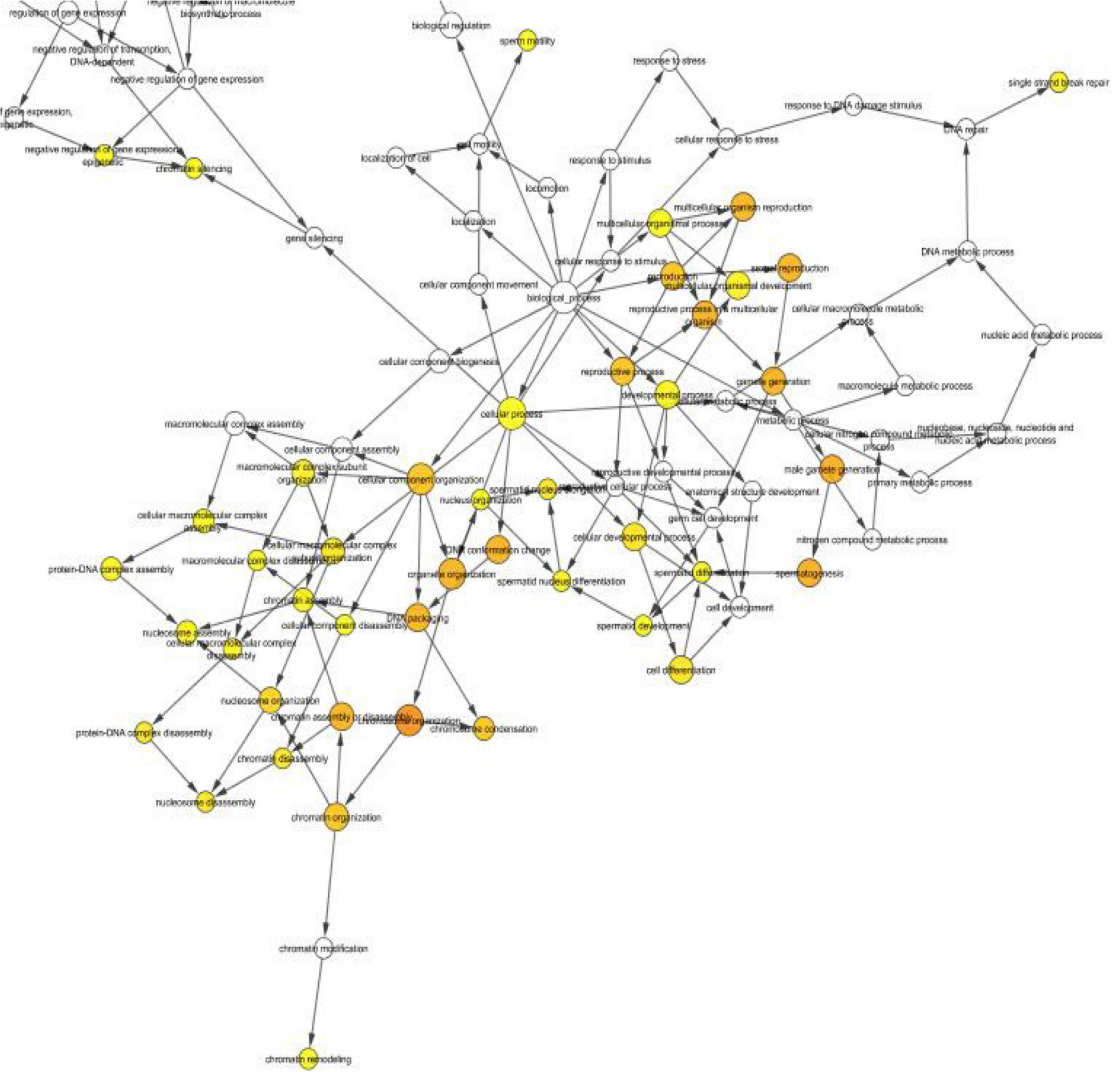
The interactome of TH2B protein with chromatin remodeling proteins. Cytoscape was used in conjunction with large protein- protein databases, understanding protein-DNA and genetic interactions. The circles represent the genes, links represent the interactions, edges depict protein-protein interactions. The bigger circle indicates the significance of expression of that gene for the given process involved. The colors represent the involvement in same function or process

The interactome networking of bovine TH2B with nuclear proteins (H2B, H2A, H3, H4 TNP1, TNP2, PRM1, BRD4 and CHD5) showed the significant gene ontology overexpression terms (*P* < 0.05; Table. 3). We showed that TH2B is involved in male gamete generation, chromosome organization, spermatogenesis, gamete generation, DNA packaging, DNA conformation change, chromatin organization, reproductive process, nucleosome organization, chromatin disassembly, spermatid nucleus elongation, nucleosome assembly, protein-DNA complex disassembly, spermatid nucleus differentiation, sperm motility, chromatin organization, chromatin condensation, single strand break repair, chromatin assembly, chromatin silencing, nucleus organization, spermatid development, spermatid differentiation, and chromatin remodeling (Fig. 8).

**Table 3:** Gene Ontology (GO) overexpression terms for *TH2B* in bull. The interactome of *TH2B* with *H2B, H2A, H3, H4 TNP1, TNP2, PRM1, BRD4* and *CHD5* was generated using BiNGO of Cytoscape 3.3

GO-ID	Description	Corrected-*p*-value	*P*-value
48,232	Male gamete generation	1.16E-05	<0.0001
51,276	Chromosome Organization	7.92E-07	<0.0001
7283	Spermatogenesis	1.16E-05	<0.0001
7276	Gamete generation	2.21E-05	<0.0001
6323	DNA packaging	2.43E-05	<0.0001
71,103	DNA conformation change	2.43E-05	<0.0001
6333	Chromatin assembly or disassembly	2.43E-05	<0.0001
6325	Chromatin organization	1.35E-04	<0.0001
22,414	Reproductive process	1.35E-04	<0.0001
30,261	Chromatin condensation	1.59E-04	<0.0001
34,728	Nucleosome organization	6.08E-04	<0.0001
31,498	Chromatin disassembly	4.12E-03	0.00412
7290	Spermatid nucleus elongation	4.12E-03	0.00412
6337	Nucleosome assembly	4.12E-03	0.00412
32,986	Protein-DNA complex disassembly	4.12E-03	0.00412
12	Single strand break repair	1.36E-02	0.0136
7289	Spermatid nucleus differentiation	1.36E-02	0.0136
6334	Nucleosome assembly	1.39E-02	0.0139
31,497	Chromatin assembly	1.39E-02	0.0139
30,317	Sperm motility	2.13E-02	0.0213
6338	Chromatin remodeling	2.78E-02	0.0278
6342	Chromatin silencing	2.78E-02	0.0278
6997	Nucleus organization	2.88E-02	0.0288
7286	Spermatid development	3.50E-02	0.0350
48,515	Spermatid differentiation	3.63E-02	0.0363

## Discussion

We conducted the present study to test the hypothesis that different cellular intensities, expression levels, and localization of TH2B are associated with sperm chromatin dynamics and bull fertility. To test such hypothesis, we used immunocytochemistry, Western blotting, flow cytometry and bioinformatic approaches.

Our study presents evidence that bovine TH2B was localized in the sperm head. The intensity of TH2B in sperm from high fertility bulls decreased as fertility increased (*P* = 0.018). The expression levels of TH2B decreased in sperm from high fertility bulls as fertility score increased (*P* = 0.0055) and increased in low fertility bulls but was not significant (*P* = 0.0737). Bovine TH2B and H2B sequences were highly similar (85.71%), as revealed by percent identity matrix (PIM) score. The H2B Emboss dotmatcher showed that the predicted bovine TH2B sequence is similar to TH2B of human, which has few possible frame shift mutation sites. Bovine H2B and human H2B have a remarkably perfect diagonal line match, revealing that H2B is highly conserved. STRING database [33]) was employed to provide a critical assessment, specific protein-protein interactions of bovine TH2B and sperm nuclear proteins, and comparisons with human and mouse proteins.

Since TH2B plays an important role in eviction of canonical histones and replacement of TNPs with protamines in the mouse [6], disruption of *th2b* gene in the mouse resulted in absence of TH2B protein, causing sterility in mouse [19]. However, it was unclear to assign which testis specific histone is responsible for this paradigm shift. Our results suggest that, in the bovine, such histone is TH2B. The interactome of TH2B revealed that TH2B influences male gamete generation, chromosome organization, spermatogenesis, gamete generation, DNA packaging, DNA conformation change, chromatin organization, reproductive process, nucleosome organization, chromatin disassembly, spermatid nucleus elongation, nucleosome assembly, protein-DNA complex disassembly, spermatid nucleus differentiation, sperm motility, chromatin organization, chromatin condensation, single strand break repair, chromatin assembly, chromatin silencing, nucleus organization, spermatid development, spermatid differentiation, and chromatin remodeling with significant GO terms. All of these functions had not been known for bovine TH2B and hence we generated new knowledge on understanding chromatin dynamics and functions regulated by TH2B.

Our results shed light on the role of TH2B in shaping bovine sperm chromatin and interacting with chromatin remodeling proteins BRD4 and CHD5. The intensity as well as expression levels of TH2B were significantly lower in sperm from high fertility group, paving the way for increased incorporation of protamines into the matured sperm chromatin resulting into increased number of healthy sperm. However, this trend was completely reversed in low fertility group; higher TH2B retention resulting in less protamine incorporation into chromatin suggesting loosening of sperm chromatin and higher numbers of immature sperm. Another possibility is that eviction of the canonical histones by TH2B might be hindering the protamine incorporation to a certain level because of abnormal retention TH2B. This is consistent with the findings of Dogan et al. (2015) [27], who showed that sperm from high fertile bulls have higher protamine levels than those from low fertile bulls resulted in less amounts of TH2B in HF and increased retention of TH2B in LF bulls.

The sample size in our immunocytochemistry analysis limited the statistical power needed to detect significant differences between the groups of bulls with high and low fertility. In addition, TH2B fluorescence signal was faint in the sperm tail, probably because TH2B was not involved with sperm chromatin [26]. In this study, we used predicted gene and protein sequences for bovine TH2B (http://www.ncbi.nlm.nih.gov/nuccore/XM_010825421.2) because the actual bovine sequences have not yet been reported to NCBI. However, an 85% homology between human sperm TH2B and somatic H2B has been clearly demonstrated through laboratory experiments [7]. Moreover, the homology between predicted bovine TH2B and H2B used in our computational analysis was 85.7%, signifying the similar proportion of changes in H2B to TH2B and possibly similar kind of roles in bovines.

In the bull, sperm chromatin dynamics was thought to be controlled by replacement of histones by protamines. This is a multi-step process involving chromatin remodelers BRD4, CHD5 and TH2B with a scope of many additional nuclear chromatin associated proteins. Therefore, the mechanisms of histone to protamine replacement are multi-cascade reactions, in which different chromatin remodelers such as BRD and CHD family proteins are involved in loosening the DNA nuclear protein binding in sperm chromatin. The results presented in our study advance the understanding of the role of TH2B in remodeling sperm chromatin dynamics.

## Conclusions

Presence of TH2B variant in bull sperm initiates replacement of histones by TNPs and then by protamines, all of which are in accordance with the prior findings about the function of TH2B in the mouse [6, 8, 19]. Our results suggest the role of TH2B in facilitating the histone to protamine transformation in bovine. Recently, researchers have shown that chromatin remodelers BRD4 and CHD5 regulate histone to protamine transition in sperm chromatin in the mouse [6, 8]. Our study about bovine functional genome also suggests that the chromatin compaction and eviction of histones is dependent on all three proteins together, BRD4, CHD5 and TH2B, where TH2B may be involved in catalyzing that transformation. We analyzed TH2B networks in the chromatin of bovine sperm to depict the cellular location of TH2B variant, and to detect the TH2B protein. The results are significant because they help us better understand the mechanisms of chromatin compaction in bovine sperm and can be applicable in reproductive biotechnology in mammals including humans.

## Abbreviations

BD: Becton Dickinson
BiNGO: Biological networks gene ontology tool
BRD4: Bromodomain-containing protein 4
CA: California
CHAPS: 3-((3-cholamidopropyl) dimethylammonio)-1-propanesulfonate
CHD5: DNA binding protein 5
DNA: Deoxy ribonucleic acid
DTT: Dithiothreitol
EDTA: Ethylenediaminetetraacetic acid
FACSCalibur: Fluorescence-activated cell sorting Calibur
Fig.: Figure
FITC: Fluorescein isothiocyanate
H2B: Histone 2B
H3: Histone 3
H4: Histone 4
HCl: Hydrochloric acid
HF: High fertility
HIST1H2BA orTH2B: Testis specific histone 2B
HRP: Horseradish peroxidase
ICC: Immunofluorescence
IgG: Immunoglobulin G
Inc.: Including
LF: Low fertility
MA: Massachusetts
Min: Minute
mM: Millimolar
NC: North Carolina
nm: Nanometer
PAGE: Polyacrylamide gel electrophoresis
PBS: Phosphate buffered saline
PIM: Percent identity matrix
PMSF: Phenyl methylsulfonyl fluoride
PRM: Protamine
PROC MIXED: The mixed procedure
RT: Room temperature
SAS: *S*oftware and services
SDS: Sodium dodecyl sulfate
STRING: Search tool for the retrieval of inter-acting proteins
TCA: Trichloroacetic acid
TNP1: Transition nuclear proteins 1
TNP2: Transition nuclear proteins 2
TNPs: Transition nuclear proteins
USA: United States of America
WI: Wisconsin
